# Longitudinal change in health-related quality of life in people with prevalent and incident type 2 diabetes compared to diabetes-free controls

**DOI:** 10.1371/journal.pone.0176895

**Published:** 2017-05-03

**Authors:** Michaela Schunk, Peter Reitmeir, Ina-Maria Rückert-Eheberg, Teresa Tamayo, Sabine Schipf, Christa Meisinger, Annette Peters, Christa Scheidt-Nave, Ute Ellert, Saskia Hartwig, Alexander Kluttig, Henry Völzke, Rolf Holle

**Affiliations:** 1 Helmholtz Zentrum München, German Research Center for Environmental Health (GmbH), Institute of Health Economics and Health Care Management, Neuherberg, Germany; 2 German Center for Diabetes Research (DZD), Partner Site Helmholtz Zentrum München, Germany; 3 Helmholtz Zentrum München, German Research Center for Environmental Health (GmbH), Institute of Epidemiology II, Neuherberg, Germany; 4 Institute of Biometrics and Epidemiology, German Diabetes Center, Leibniz Center for Diabetes Research at Heinrich-Heine-University, Düsseldorf, Germany; 5 German Center for Diabetes Research (DZD), Partner Site DDZ Düsseldorf, Germany; 6 Institute for Community Medicine, Ernst Moritz Arndt-University, Greifswald, Germany; 7 German Center for Diabetes Research (DZD), Project Partner Site Uni Greifswald, Greifswald, Germany; 8 Department of Epidemiology and Health Monitoring, Robert-Koch-Institute, Berlin, Germany; 9 German Center for Diabetes Research (DZD), Project Partner Site RKI, Berlin, Germany; 10 Institute of Medical Epidemiology, Biostatistics and Informatics, Martin-Luther-University Halle-Wittenberg, Halle (Saale), Germany; 11 German Center for Diabetes Research (DZD), Project Partner Site Uni Halle-Wittenberg, Halle, Germany; 12 German Center for Cardiovascular Research, Partner Site Greifswald, Germany; Soochow University Medical College, CHINA

## Abstract

**Objective:**

The objective of this analysis is to compare people with prevalent type 2 diabetes, incident type 2 diabetes and without diabetes with respect to longitudinal change in health-related quality of life (HRQOL) when adjusting for baseline determinants of HRQOL.

**Research design and methods:**

Primary baseline and follow-up data from three regional and one national population-based cohort studies in Germany were pooled for analysis. HRQOL was measured using physical and mental health summary scores (PCS and MCS) from the German version of the Short Form Health Survey with 36 or 12 items. Mean score change per observation year was compared between the three groups (prevalent diabetes, incident diabetes, no diabetes) based on linear regression models.

**Results:**

The analysis included pooled data from 5367 people aged 45–74 years at baseline. Of these, 85.5% reported no diabetes at baseline and follow-up, 6.3% reported diabetes at both baseline and follow-up (prevalent diabetes), and 8.2% reported diabetes only at follow-up (incident diabetes). Over a mean observation period of 8.7 years, annual decline in HRQOL scores is pronounced at 0.27–0.32 (PCS) and 0.34–0.38 (MCS) in the group with prevalent diabetes compared with people without diabetes. Those with incident diabetes showed intermediate values but did not differ significantly from people without diabetes after adjustment for covariates in the full model.

**Conclusion:**

Compared with data from cross-sectional analysis, the HRQOL loss associated with prevalent diabetes appears to be much larger than previously assumed.

## Introduction

Health-related quality of life (HRQOL) is a central parameter for assessment of the burden of illness as well as the clinical effectiveness of care and treatment. Estimations of the impact of type 2 diabetes on HRQOL underline the significant burden of this complex disease with its correlates in terms of lifestyle factors, comorbidities, and diabetes complications. In population-based, cross-sectional studies, HRQOL measured with generic instruments such as the EQ-5D or the Short Form Health Survey with 36 or 12 items (SF-36/12) has been found to be at least moderately impaired in people with diabetes compared with people without diabetes [1, 2]. Studies using the SF-36/12, which measures different domains of physical and mental HRQOL, found the impairment in the physical health summary score (PCS) to be much larger than that in the mental health summary score (MCS) [3–6]. Effect sizes with regard to the PCS are considered to be clinically relevant [7]. The main drivers of the HRQOL detriment associated with diabetes are comorbidities and complications [8–12].

Longitudinal analysis adds to an understanding of the variation in HRQOL over time, but data are scarce compared with cross-sectional data. To the best of our knowledge, only three previous studies had a longitudinal design. One is a US-based survey study that used the EQ-5D as a measurement for health status and collected data in 2004 and 2009 [13]. The decline in EQ-5D index scores in people with diabetes was significantly greater and twice the size compared with people without diabetes. The other two studies are not population-based and do not include people without diabetes [14, 15]. Bayliss et al. analyzed the Medical Outcome Study data from the early 1990s and compared the impact of diabetes on HRQOL with other chronic diseases measured over 4 years [14]. Diabetes increased the odds of a decline in SF-36 PCS scores to a greater degree than other diseases except for congestive heart failure. The most recent longitudinal study, undertaken with a practice-based sample in Germany between 2000 and 2007, analyzed changes in HRQOL among general practice patients with diabetes [15]. It underlined the impact of diabetes-related complications and comorbidities on lower HRQOL scores at follow-up after 5 years. These studies suggest the benefits of longitudinal data to elucidate the true burden of diabetes, but further studies including a control group and with longer time horizons are needed to get a valid estimate of this burden.

In this study, we analyze a large dataset with pooled data from four population-based cohort studies in Germany to measure the impact of diabetes on HRQOL over an observation time of 5–12 years. The objective of this analysis is to compare people with prevalent diabetes and those with incident diabetes with people without diabetes with respect to longitudinal change in HRQOL when adjusting for baseline determinants of HRQOL.

## Research design and methods

### Study design

Baseline and follow-up data from three regional and one national population-based cohort studies were included in this analysis: the Cooperative Health Research in the Region of Augsburg (KORA) study; the Cardiovascular Disease, Living and Ageing in Halle (CARLA) Study; the Study of Health in Pomerania (SHIP); and the nationwide German National Health Interview and Examination Survey (baseline: GNHIES98; follow-up: DEGS1) carried out by the Robert Koch Institute (RKI). Data collection for baseline studies was performed between 1997 and 2006 and for follow-up examinations between 2006 and 2012 (Table 1).

**Table 1:** Studies included in the pooled analysis.

Name of study	Study periods Baseline/follow-up	Sampling	Total N (response %) Baseline/follow-up		Age range (years) of participants at baseline
Cooperative Health Research in the Region of Augsburg (KORA S4–F4)	1999–2001 7 years FU	Two-stage cluster sample	4261 (67)/	3080 (80)	25–74
Study of Health in Pomerania (SHIP 0–2)	1997–2001 11 years FU	Two-stage cluster sample	4308 (69)/	3300 (84)	20–79
CARdiovascular disease, Living and Ageing in Halle (CARLA 0–1)	2002–2006 5 years FU	Stratified random sample	1779 (64)/	1436 (86)	45–83
German National Health Interview and Examination Survey (GNHIES98–DEGS1)	1997–1999 12 years FU	Two-stage cluster sample	7124 (61)/	3795 (64)	18–79

### Participants

Study details have been described elsewhere [16–22]. All included studies are population-based cohort studies comparable with regard to study design, sampling methods, and response rates. All studies carried out baseline and follow-up assessments in local study centers, where participants were examined, laboratory testing material was collected, and standardized computer-assisted clinical medical interviews (CAPI) were conducted. In addition, participants filled in self-administered questionnaires. All participants gave written informed consent. All studies were approved by local ethics committees and monitored by external quality assurance boards.

Type 2 diabetes is defined based on self-report of physician-diagnosed diabetes mellitus or self-reported intake of oral anti-diabetic agents or insulin. Participants with an age of onset of ≤ 30 years were excluded, assuming they had type 1 diabetes. Participants with missing information on diabetes were included when they self-reported the intake of oral anti-diabetic agents but excluded when they reported the intake of insulin, again to reduce the risk of including participants with type 1 diabetes. Following this definition, participants with type 2 diabetes at neither baseline nor follow-up were classified as “no diabetes”; those with type 2 diabetes at both baseline and follow-up were classified as “prevalent diabetes”. Participants with type 2 diabetes only at follow-up were classified as “incident diabetes”. To ensure comparability between studies, analyses were restricted to participants aged between 45 and 74 years at baseline. Only participants with data from both baseline and follow-up were included in this study.

**Fig 1:**
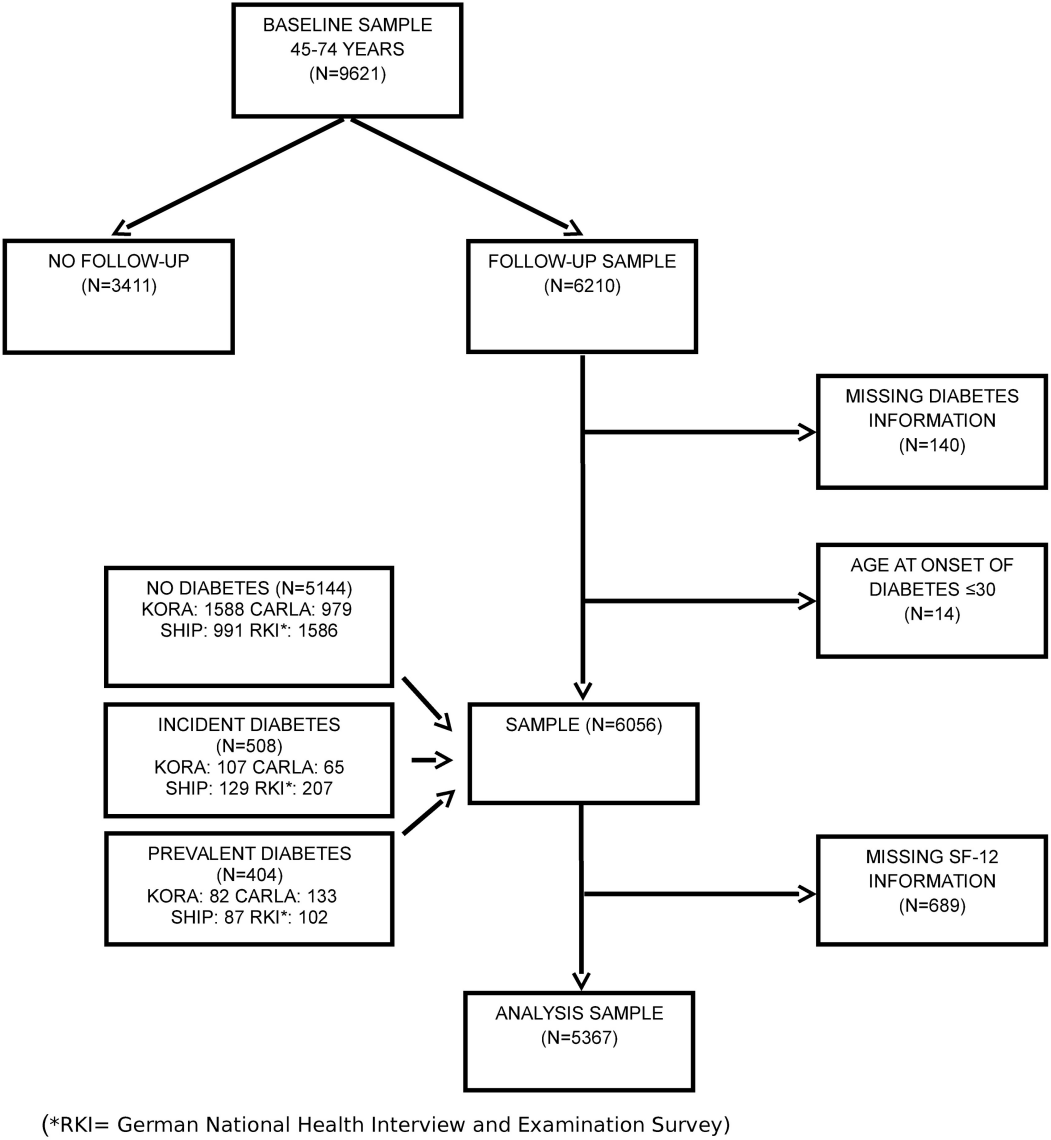
Flowchart of sample.

The flowchart of the sample by diabetes status and study is illustrated in Fig 1.

### Variables

The outcome variable in this study is HRQOL, as measured with a generic instrument, the German version of the Short Form Health Survey with 36 or 12 items (SF-36/12) [23]. This instrument was applied across all studies as a self-administered questionnaire, except for the KORA baseline study where it was part of the clinical medical interview. Two SF12/36 summary scores will be used for this analysis: the physical component summary (PCS) and the mental component summary (MCS). KORA, SHIP, and CARLA used version 1 of the SF-12 (SF-12v1) in baseline and follow-up studies; the GNHIES98 survey used version 1 of the SF-36 (SF-36v1), but DEGS1 used version 2 (SF-36v2) [24]. Scores are calculated using standard algorithms [25]. As reported in the literature, SF-12v1 and SF-36v1 summary scores express a high degree of correspondence [25, 26]. To compare the summary scores across studies, we used the US 1998 norm data and transformed the SF36v2 values to SF36vs1 values, as allowed for in the scoring software [27]. Both scores are transformed to a mean of 50 and standard deviation of 10, with higher values indicating better HRQOL.

Other variables that were controlled for included age (in years), sex, and study. Furthermore, socioeconomic information, comorbidities, and lifestyle variables assessed at baseline were included. Education was treated as a dichotomous variable with two levels (< 10 years vs. ≥ 10 years). Income was transformed to equivalent income and categorized with reference to the median income of the GNHIES98 study, irrespective of when the baseline assessment took place exactly in each study. Living alone (yes vs. no) was based on information regarding household size. Information on comorbidities followed survey questions if myocardial infarction or stroke had ever been diagnosed or treated in hospital, and if a doctor had ever diagnosed hypertension or hyperlipidemia. Weight and height were measured at the study center and calculated as body mass index (BMI; weight in kilograms divided by the squared height in meters), classified in six groups according to the WHO (underweight: BMI <18.5 kg/m^2^; normal weight: 18.5≤BMI<25; pre-obesity: 25≤BMI<30; moderate obesity (i.e., obesity class 1): 30≤BMI<35; severe obesity (i.e., obesity class 2): 35≤BMI<40; very severe obesity (i.e., obesity class 3): BMI≥40 kg/m^2^) [28]. Lifestyle variables included physical activity (<1 hour per week vs. more), alcohol consumption (>40g/day for men; >20g/day for women vs. less), and smoking (current vs. formerly or never).

Primary data from the studies were added to a joint database. All variables were recoded following standard procedures, established jointly with co-workers from the DIAB-CORE consortium [29–31].

### Missing values

Participants with any missing values in the SF-12 items were excluded because the calculation of summary scores is not possible in this case, following the recommendations of the instrument’s authors [25]. Exclusions applied to N = 136 (KORA, 7.7% of sample), N = 118 (CARLA; 10.0% of sample), and N = 191 (SHIP, 15.8% of sample). In studies that used the SF-36, participants were excluded only when missing values did not allow for calculation of the subscales [32] (N = 244, RKI; 12.9% of sample). Taken together, N = 689 (11.4%) of the pooled sample were excluded due to missing values in the SF-12v1 and SF-36v1. Final sample size for the analysis was N = 5367 (Table 2 and Fig 1). Participants with missing values in covariates were excluded (N = 65) in the respective regression models.

**Table 2:** Sample description.

	No diabetes	Incident diabetes	Prevalent diabetes	Total
N	4588 (85.5)	441 (8.2)	338 (6.3)	5367 (100.0)
Sex, female (%)	50.9	45.8	41.7	49.9
Age at baseline (years, mean (SD))	56.8 (7.7)	58.4 (7.6)	60.7 (7.1)	57.2 (7.7)
Study				
KORA	1471 (32.1)	99 (22.4)	71 (21.0)	1641 (30.6)
CARLA	885 (19.3)	61 (13.8)	113 (33.4)	1059 (19.7)
SHIP	833 (18.2)	109 (24.7)	74 (21.9)	1016 (18.9)
RKI	1399 (30.5)	172 (39.0)	80 (23.7)	1651 (30.8)
HRQOL baseline				
PCS	48.5 (8.9)	46.7 (9.1)	44.2 (10.0)	48.1 (9.1)
MCS	51.9 (8.9)	52.3 (9.1)	51.6 (10.4)	51.9 (9.0)
Education (<10 years)	2457 (53.6)	281 (63.7)	233 (68.9)	2971 (55.4)
Equivalent income of GNHIES 98 median income				
less than 60%	437 (9.5)	45 (10.2)	35 (10.4)	517 (9.6)
between median income and 60%	1265 (27.6)	148 (33.6)	126 (37.3)	1539 (28.7)
between median income and 150%	1553 (33.8)	143 (32.4)	118 (34.9)	1814 (33.8)
more than 150% above	975 (21.3)	62 (14.1)	39 (11.5)	1076 (20.0)
Living alone	715 (15.6)	66 (15.0)	59 (17.5)	840 (15.7)
Smoking (current)	875 (19.1)	110 (24.9)	50 (14.8)	1035 (19.3)
Alcohol (>40g/day ♂; >20g/day ♀)	576 (12.6)	50 (11.3)	22 (6.5)	648 (12.1)
Physical activity (less than 1 hour/week)	2057 (44.8)	255 (57.8)	214 (63.3)	2526 (47.1)
BMI group				
BMI <18.5 kg/m^2^	7 (0.2)	0 (0.0)	0 (0.0)	7 (0.1)
BMI ≥18.5 and ≤24.9 kg/m^2^	1285 (28.0)	32 (7.3)	24 (7.1)	1341 (25.0)
BMI ≥25.0 and ≤29.9 kg/m^2^	2223 (48.5)	161 (36.5)	127 (37.6)	2511 (46.8)
BMI ≥30.0 and ≤34.9 kg/m^2^	847 (18.5)	163 (37.0)	117 (34.6)	1127 (21.0)
BMI ≥35.0 and ≤39.9 kg/m^2^	176 (3.8)	57 (12.9)	52 (15.4)	285 (5.3)
BMI ≥40.0 kg/m^2^	46 (1.0)	26 (5.9)	17 (5.0)	89 (1.7)
Myocardial infarction	126 (2.7)	21 (4.8)	27 (8.0)	174 (3.2)
Stroke	70 (1.5)	14 (3.2)	16 (4.7)	100 (1.9)
Hypertension	1755 (38.3)	269 (61.0)	240 (71.0)	2264 (42.2)
Hyperlipidemia	1348 (29.4)	195 (44.2)	155 (45.9)	1698 (31.6)

### Statistical analysis

Descriptive statistics of the sample were reported separately for those with prevalent diabetes, incident diabetes, and without diabetes. To examine the diabetes-related impact on the course of HRQOL, the absolute change in PCS or MCS scores per observation year (difference between the baseline and follow-up score divided by the time difference between both examination time points) was chosen as the main analysis parameter. Comparisons between the three groups (prevalent diabetes, incident diabetes, no diabetes) were done by contrasts based on linear regression models. We first used a crude regression model with adjustment for age, sex, and study (model 1). In a second model we additionally adjusted for baseline values of covariates such as socioeconomic variables (school, income), living alone, smoking, alcohol consumption, physical activity, and BMI (model 2), and in a third model also comorbidities such as myocardial infarction, stroke, hypertension and hypercholesterolemia were included (model 3). All models were applied separately to PCS and MCS.

All estimates were reported with 95% confidence intervals (95%CI). Statistical significance was assumed with P-values less than 0.05. All analyses were performed using SAS statistical software version 9.3. (SAS Institute, Cary, NC, USA).

Furthermore, we performed several sensitivity analyses. Interactions between diabetes status and sex, the impact of adjusting the models for baseline PCS and baseline MCS, respectively, and whether coding of age as a categorical variable leads to differences in the size and direction of changes in scores were tested.

## Results

### Participants

The analysis includes pooled data from 5367 people aged 45–74 years at baseline from three regional population-based cohort studies in Germany, N = 1641 (30.6%) in the South (KORA), 1016 (18.9%) in the North (SHIP), and 1059 (19.7%) in the North-east (CARLA), as well as 1651 (30.8%) from a national cohort study (GNHIES98/DEGS1; RKI). Of these, 85.5% report no diabetes at baseline and follow-up, 6.3% report diabetes at both baseline and follow-up (prevalent diabetes), and 8.2% report diabetes only at follow-up (incident diabetes). Studies differ in the proportion of participants with prevalent, incident and no diabetes. A greater proportion of participants were classified with prevalent diabetes in the CARLA study and with incident diabetes in the DEGS1study, compared with the other studies. Generally, the two studies with longer follow-up times (SHIP, 11 years; GNHIES98/DEGS1, 12 years) contribute more incident cases than the studies with shorter follow-up times. Mean follow-up time is 8.7 years (min. 3.4 years; max. 14.2 years) for participants with no diabetes, 9.6 years (min. 3.7 years; max. 14.3 years) for participants with incident diabetes, and 8.1 years (min. 3.8 years; max. 14.1 years) for participants with prevalent diabetes.

Descriptive data on variables of interest at baseline in total and by diabetes subgroup are shown in Table 2.

### Main results

**Fig 2:**
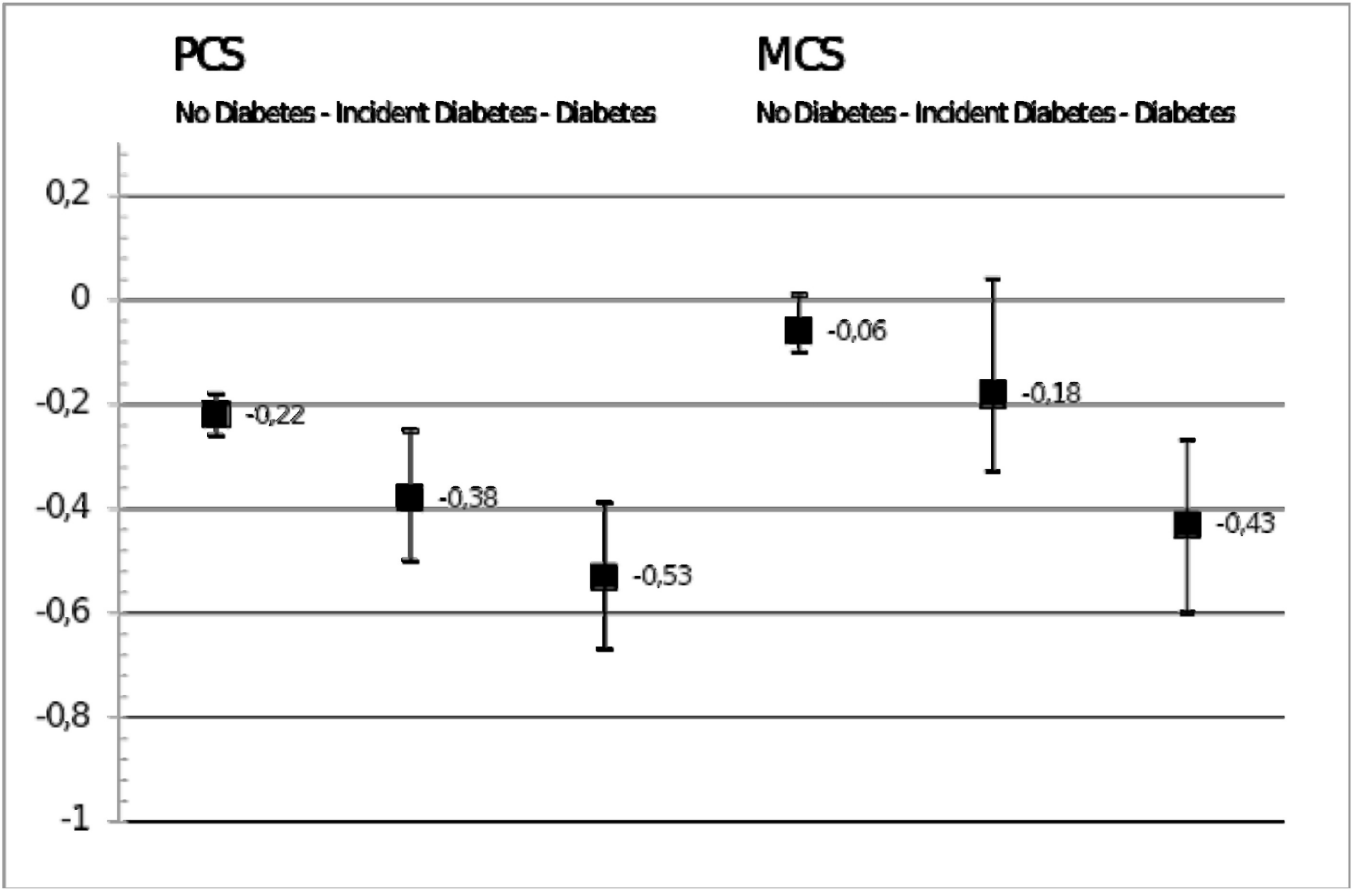
Mean score change per observation year, adjusted for age, sex and study.

Compared to mean score changes among adults without diabetes as the reference group, significantly larger declines in mean PCS scores are observed among adults with prevalent as well as among those with incident diabetes (Table 3, model 1). Mean score change in PCS per observation year, adjusted for age and study, as shown in Fig 2, is –0.22 (–0.26; –0.18) points, –0.38 (–0.50; –0.25) points, and –0.53 (–0.67; –0.39) points for the groups without diabetes, incident diabetes, and prevalent diabetes respectively. For MCS, the corresponding Figs are –0.06 (–0.10; 0.01), –0.18 (–0.33; –0.04), and –0.43 (–0.60; –0.27).

**Table 3:** Regression models for annual change in HRQOL scores.

Variables		Physical component summary score (PCS)			Mental component summary score (MCS)		
		Model 1	Model 2	Model 3	Model 1	Model 2	Model 3
		N = 5367	N = 5302	N = 5302	N = 5367	N = 5302	N = 5302
Diabetes	No diabetes	Ref.	Ref.	Ref.	Ref.	Ref.	Ref.
	Prevalent diabetes	**–0.32**	**–0.27**	**–0.27**	**–0.38**	**–0.35**	**–0.34**
		–0.46 to –0.17	–0.43 to –0.12	–0.43 to –0.12	–0.55 to –0.21	–0.52 to –0.17	–0.52 to –0.16
	Incident diabetes	**–0.16**	–0.11	–0.11	–0.13	–0.07	–0.07
		–0.29 to –0.03	–0.25 to 0.03	–0.25 to 0.02	–0.28 to 0.02	–0.23 to 0.08	–0.23 to 0.09
Age (in years)		**–0.013**	**–0.013**	**–0.013**	**–0.010**	**–0.010**	**–0.010**
		–0.017 to –0.008	–0.018 to –0.008	–0.018 to –0.008	–0.015 to –0.005	–0.016 to –0.004	–0.016 to –0.004
Sex (Ref. male)	Female	0.05	0.07	0.05	0.02	0.02	0.02
		–0.02 to 0.12	–0.01 to 0.14	–0.02 to 0.13	–0.06 to 0.10	–0.07 to 0.10	–0.07 to 0.10
Study	RKI	Ref.	Ref.	Ref.	Ref.	Ref.	Ref.
	CARLA	–0.09	–0.10	–0.10	–0.11	–0.13	–0.12
		–0.19 to 0.02	–0.21 to 0.01	–0.22 to 0.01	–0.23 to 0.01	–0.25 to 0.00	–0.24 to 0.01
	KORA	0.08	0.06	0.07	0.07	0.06	0.07
		–0.01 to 0.17	–0.03 to 0.16	–0.03 to 0.17	–0.03 to 0.18	–0.05 to 0.17	–0.05 to 0.18
	SHIP	0.05	0.04	0.04	**0.21**	**0.22**	**0.23**
		–0.06 to 0.15	–0.07 to 0.15	–0.07 to 0.15	0.09 to 0.33	0.10 to 0.34	0.10 to 0.35
Education (Ref. (≥10 years)	<10 years in education		0.04, –0.04 to 0.12	0.04, –0.04 to 0.12		–0.02, –0.11 to 0.07	–0.03, –0.11 to 0.06
Equivalent income (of GNHIES 98 median income)	150% above		Ref.	Ref.		Ref.	Ref.
	Between median and 150% above		–0.01, –0.12 to 0.09	–0.01, –0.12 to 0.09		–0.10, –0.22 to 0.02	–0.10, –0.22 to 0.02
	Between median and 60% below		–0.07, –0.18 to 0.05	–0.06, –0.17 to 0.05		–0.07, –0.20 to 0.06	–0.06, –0.19 to 0.07
	Less than 60%		–0.15	–0.14		–0.16	–0.16
			–0.30 to 0.01	–0.29 to 0.02		–0.34 to 0.01	–0.33 to 0.01
Living alone			**–0.13**	**–0.14**		**0.18**	**0.17**
(Ref.: no)			–0.23 to –0.02	–0.24 to –0.03		0.06 to 0.30	0.05 to 0.30
Smoking (Ref.: formerly or never)	Current		**–0.10**	**–0.10**		–0.01	–0.01
			–0.19 to 0.00	–0.19 to 0.00		–0.12 to 0.10	–0.12 to 0.10
Alcohol (Ref.: less)	>40g/day ♂;		–0.08	–0.08		–0.09	–0.09
	>20g/day ♀		–0.19 to 0.03	–0.20 to 0.03		–0.22 to 0.04	–0.22 to 0.04
Phys. activity (Ref.: more)	Less than 1 hour/week		–0.02, –0.09 to 0.06	–0.02, –0.09 to 0.06		–0.02, –0.10 to 0.07	–0.01, –0.10 to 0.07
BMI group	<18.5 kg/m^2^		0.27	0.27		0.04	0.05
			–0.72 to 1.26	–0.72 to 1.26		–1.09 to 1.17	–1.08 to 1.18
	≥18.5 and ≤24.9 kg/m^2^		Ref.	Ref.		Ref.	Ref.
	≥25.0 and ≤29.9 kg/m^2^		–0.04	–0.05		0.02	0.02
			–0.13 to 0.05	–0.14 to 0.05		–0.09 to 0.12	–0.09 to 0.12
	≥30.0 and ≤34.9 kg/m^2^		**–0.17**	**–0.17**		–0.12	–0.11
			–0.28 to –0.06	–0.28 to –0.06		–0.25 to 0.01	–0.24 to 0.02
	≥35.0 and ≤39.9 kg/m^2^		–0.01	–0.01		–0.11	–0.10
			–0.18 to 0.17	–0.19 to 0.17		–0.31 to 0.09	–0.30 to 0.11
	≥40.0 kg/m^2^		**–0.47**	**–0.47**		–0.12	–0.10
			–0.76 to –0.18	–0.76 to –0.18		–0.45 to 0.22	–0.43 to 0.23
Myocardial infarction				**–0.30**			–0.09
				–0.51 to –0.09			–0.33 to 0.14
Stroke				–0.01			–0.03
				–0.28 to 0.26			–0.34 to 0.27
Hypertension				0.01			–0.05
				–0.07 to 0.09			–0.14 to 0.04
Hyperlipid-emia				0.06			0.04
				–0.02 to 0.14			–0.05 to 0.13

Bold-faced numbers are statistically significant with nominal P<0.05.

Differences in mean score changes are statistically significant for prevalent diabetes vs. no diabetes for PCS and MCS (both P-values <0.0001), as well as between incident diabetes and no diabetes for PCS (P-value 0.018), and between incident diabetes and prevalent diabetes for MCS (P-value 0.024). Significantly larger declines in mean MCS scores compared to the reference group were restricted to persons with prevalent diabetes. Changes in mean MCS scores significantly differed between incident diabetes and prevalent diabetes.

Results relating to the comparison of persons with and without diabetes at baseline largely persisted when additionally controlling for socioeconomic differences and lifestyle variables and comorbidities (Table 3, model 2 and model 3). In contrast, differences in mean change of PCS scores between persons with incident diabetes and the reference groups were explained in models adjusting for additional covariables.

Mean summary scores in PCS and MCS decreased with increasing age (Table 3). MCS scores are associated with study effects, with the SHIP study displaying less change per annum than the other studies. Living alone, smoking, obesity, and myocardial infarction is associated with even larger negative annual changes in PCS scores. No risk factors are associated with MCS scores except for living alone, showing a positive relationship. Thus, living alone is reversely related to PCS and MCS scores.

### Sensitivity analyses

Interactions between diabetes status and sex were tested but showed no association with change in HRQOL. Categorization of the variable age did not influence the results. In a sensitivity analysis, we adjusted for baseline PCS and MCS scores in the regression models. This led to even more pronounced effects of diabetes on change scores in the same direction. An additional analysis stratified by study showed no heterogeneity in the effect of diabetes status on annual change in PCS and MCS.

## Discussion

This analysis shows that, in longitudinal perspective over a mean observation time of 8.7 years, annual decline in HRQOL scores in the group with prevalent diabetes is pronounced at 0.53 (PCS) and 0.43 (MCS) and significantly larger than in people without diabetes. Those with incident diabetes showed intermediate values but did not differ significantly from people without diabetes after adjustment for covariates in the full model. Effect sizes remain stable when adjusting for socioeconomic differences and risk factors as well as comorbidities. Living alone, smoking, obesity, and a history of myocardial infarction are associated with more pronounced decline in PCS scores.

The only other longitudinal study comparing people with and without diabetes used the EQ-5D index score, but similarly found the decline in people with diabetes to be twice the size of the decline in people without diabetes over a measurement period of 5 years [13]. Unlike our analysis, Grandy et al. did not control for comorbidities and risk factors. However, our data using the SF-12 as a generic HRQOL instrument confirm these results. Both EQ-5D and SF-12 are widely used, with fewer ceiling effects and a greater breadth to cover mental health dimensions as advantages of the SF-12 [33].

How does the diabetes-related trend in HRQOL shown in this longitudinal study compare with data from cross-sectional analysis? Our previous cross-sectional analysis of baseline data from the same studies as those analyzed here found a PCS decrease of less than 2 points per age decade, both for people without diabetes and, at a lower starting position, for people with prevalent diabetes [3]. However, our present longitudinal analysis shows a much faster decline in PCS in people with prevalent diabetes (about 5 points over 10 years) because we follow individual trajectories of prevalent and incident diabetes cases in a longitudinal perspective.

For MCS in cross-sectional analysis, there is no decline in the age groups 45–74 years, neither for people with diabetes nor for those without diabetes [3]. This longitudinal analysis shows a decline in MCS over time, which is statistically significant only for people with prevalent diabetes. Arguably, as the age groups included in our study now go beyond 74 years (maximum follow-up time was 14 years), a decline in MCS scores may be more visible than in studies that do not include very old people [34].

A longitudinal study of a practice-based diabetes sample on predictors of change in HRQOL, which also used PCS and MCS scores as outcome variables [15], found age, smoking, diabetes complications, and BMI associated with PCS decline. For a decline in MCS, diabetes complications and self-reported diagnosis of depression were identified as associated with HRQOL changes. In our analysis living alone was the only risk factor associated with both PCS and MCS scores. Interestingly, the effects were reversed, negative for PCS and positive for MCS. Further studies of a broader set of variables measuring functional and psychological well-being in relation to chronic disease, living arrangements and social support are needed [35].

Likewise, in other studies including our own cross-sectional study, the diabetes effect on MCS scores was more pronounced in women than in men [36]. In a longitudinal perspective, however, no gender difference was found with regard to PCS or MCS scores.

Some limitations of this analysis have to be discussed. First, with only two measurement points, an analysis of longitudinal change gives less stable results than with multiple waves of data [37]. Follow-up duration, which in our study extended from 5 to 12 years for the different cohorts, has an influence on the estimation of annual change and on the comparability of incident cases. We therefore adjusted all our analyses for study cohort. Although all four cohorts draw on representative samples to the respective region (or across Germany, as in the case of the GNHIES98/DEGS1 studies), our data is affected by selection bias. Surveys tend to under-represent very wealthy and very poor segments of the population as well as institutionalized people [38], possibly for those with diabetes and for those without diabetes. People who died between baseline and follow-up were regarded in the same way as drop-outs due to other causes. This may introduce a healthy survivor bias to our results. Moreover, ill health often leads to study drop-out and may affect those with diabetes more. Thus, effect sizes between the groups in our study may be underestimated.

Most data used here draw on self-report. The classification of diabetes, risk factors, and comorbidities was based upon self-report rather than clinical measures (with the exception of BMI which was measured at the study center). Studies have shown that the misclassification error due to self-reported information on such variables is relatively small [39, 40]. Because of the restrictions of a commonly defined data pool, information on diabetes complications as well as other comorbidities such as depression, which contribute to overall health burden, were not collected across all studies and thus could not be analyzed. Studies also used different versions of the SF12/SF36, thus our analysis relies on the comparability of the summary scores as assured in the scoring manual [27].

Our objective was the comparison of HRQOL in people with and without diabetes. For this task, only a generic HRQOL instrument such as the validated and widely used SF-36/12 should be used. However, the diabetes burden can only be partly captured by generic HRQOL instruments and needs to be augmented by studies with diabetes-specific instruments [41, 42]. Further studies of individual trajectories in HRQOL over time as well as benchmarks for the interpretation of score differences are needed [37, 43].

In conclusion, we provide important longitudinal data to estimate the incremental burden of diabetes with a validated instrument and a large pooled dataset combining individual data from four population-based cohort studies. Studies were similar regarding study design and sampling frame, providing a population-based reference. Our analysis indicates that the diabetes-associated loss in HRQOL is much larger in the longitudinal perspective than assumed from cross-sectional analysis. Therefore, efforts to improve diabetes management, including evidence based treatment and advice for self-management, are key to alleviate the diabetes burden to afflicted patients.

## Supporting information

S1 File Survey questions. (DOC)
