# Supplementary material for: Longitudinal change in health-related quality of life in people with prevalent and incident type 2 diabetes compared to diabetes-free controls
Source: PLoS One. 2017 May 3;12(5):e0176895. doi: 10.1371/journal.pone.0176895 (PMC5415190; doi:10.1371/journal.pone.0176895)
Supplement: S1 File — (DOC) [file pone.0176895.s001.doc]

**Copy of survey questions**

|  | German | English |
| --- | --- | --- |
| self-report of physician-diagnosed diabetes mellitus | Wurde bei Ihnen eine Diabetes Erkrankung festgestellt? | Have you been diagnosed with diabetes? |
| self-reported intake of anti-diabetic medication | Wie werden Sie behandelt? | Type of treatment for diabetes? |
| age of onset of diabetes | Geben Sie das Jahr an, in dem das festgestellt wurde | Year of diagnosis? |
| Short Form Health Survey 36/12 items | *licenced under https://www.hogrefe.de/* | *licenced under http://www.optum.ca/life-sciences/develop-evidence/patient-insights/health-surveys.html* |
| education | Welches ist Ihr höchster Schulabschluß? | Which ist your highest school-leaving qualification? |
| living alone | Leben Sie mit Ihrem Ehepartner bzw. einem Partner zusammen, gemeint ist ein gemeinsamer Haushalt? | Are you living with someone, such as a spouse or partner, in a joint household ? |
| income | Wie hoch ist das monatliche Nettoeinkommen Ihres Haushaltseinkommen, d.h. das Einkommen, das alle Haushaltsmitglieder nach Steuern und Sozialabgaben haben? | What is your monthly net disposable household income, i.e. the income all household members have after deductions for tax, social security contributions? |
| MI | Hatten Sie schon jemals einen von einem Arzt festgestellten Herzinfarkt? | Have you ever had a heart attack, diagnosed by a physician? |
| stroke | Hatten Sie schon jemals einen von einem Arzt festgestellten Schlaganfall? | Have you ever had a stroke, diagnosed by a physician? |
| hypertension | Jemals von Arzt erhöhter Blutdruck festgestellt/mitgeteilt worden? | Have you ever had hypertension, diagnosed/confirmed by a physician? |
| hyperlipidemia | Jemals von Arzt erhöhter Cholesterinspiegel festgestellt/mitgeteilt worden? | Have you ever had an elevated level of cholesterol, diagnosed/confirmed by a physician? |
| BMI based on weight and height | Was ist Ihre Körpergröße?  Was ist ihr Gewicht? | What is your body weight?  What is your body height? |
| physical activity | Wieviele Stunden pro Stunde treiben Sie Sport ? | How many hours per week are you physically active? |
| alcohol consumption | Wieviele alkoholische Getränke haben Sie in der letzten Woche zu sich genommen? | How many alcoholic beverages did you drink during the last week? |
| smoking | Rauchen Sie?  Wann habe Sie aufgehört zu Rauchen? | Do you smoke?  When did you stop smoking? |
